# Newer Gene Editing Technologies toward HIV Gene Therapy

**DOI:** 10.3390/v5112748

**Published:** 2013-11-14

**Authors:** N. Manjunath, Guohua Yi, Ying Dang, Premlata Shankar

**Affiliations:** Center of Excellence in Infectious Disease, Department of Biomedical Sciences, Paul L. Foster School of Medicine, Texas Tech University Health Sciences Center, El Paso, TX 79905, USA; E-Mails: g.yi@ttuhsc.edu (G.Y.); ying.dang@ttuhsc.edu (Y.D.)

**Keywords:** zinc finger nuclease, transcription activator-like effector nuclease, CRISPR/Cas 9, gene editing, HIV-1 therapy, CCR5 disruption

## Abstract

Despite the great success of highly active antiretroviral therapy (HAART) in ameliorating the course of HIV infection, alternative therapeutic approaches are being pursued because of practical problems associated with life-long therapy. The eradication of HIV in the so-called “Berlin patient” who received a bone marrow transplant from a CCR5-negative donor has rekindled interest in genome engineering strategies to achieve the same effect. Precise gene editing within the cells is now a realistic possibility with recent advances in understanding the DNA repair mechanisms, DNA interaction with transcription factors and bacterial defense mechanisms. Within the past few years, four novel technologies have emerged that can be engineered for recognition of specific DNA target sequences to enable site-specific gene editing: Homing Endonuclease, ZFN, TALEN, and CRISPR/Cas9 system. The most recent CRISPR/Cas9 system uses a short stretch of complementary RNA bound to Cas9 nuclease to recognize and cleave target DNA, as opposed to the previous technologies that use DNA binding motifs of either zinc finger proteins or transcription activator-like effector molecules fused to an endonuclease to mediate sequence-specific DNA cleavage. Unlike RNA interference, which requires the continued presence of effector moieties to maintain gene silencing, the newer technologies allow permanent disruption of the targeted gene after a single treatment. Here, we review the applications, limitations and future prospects of novel gene-editing strategies for use as HIV therapy.

## 1. Introduction

With the development of anti-retroviral therapy, HIV-1 infection is now manageable as a chronic disease. However, HIV-1 still remains a global epidemic responsible for considerable morbidity and mortality. Although highly active antiretroviral therapy (HAART) is effective in suppressing viral replication and reducing viral loads in HIV patients, it has limitations including high cost, patient compliance and side effects of long-term therapy, as well as emergence of drug resistance [1,2]. Moreover, although HARRT extends the lives of HIV-1 infected patients, it does not offer a permanent cure, since interruption of therapy leads to rapid rebound of viremia from latent reservoirs [3,4]. Therefore, there is a need to develop more effective countermeasures for HIV infection. 

Gene therapy is an attractive method to derive HIV-resistant cells [5,6]. Interest in this field began following the demonstration that CCR5 is a major coreceptor for HIV. Naturally occurring homozygous CCR5-Δ32 mutation (32 bp deletion in the single coding exon of the gene), which results in a frame-shift mutation that disrupts CCR5 expression on the cell surface [7,8,9], confers resistance to HIV infection. The remarkable success in eradicating HIV in the so-called “Berlin Patient” has led to a resurgence of interest in gene therapy. Here, a HIV positive patient with lymphoma, who had been transplanted with bone marrow from a CCR5-Δ32 homozygous donor, became cured with no demonstrable virus even 5 years after transplantation, showing the potential benefits of CCR5 disruption [10,11]. However, due to the low frequency of CCR5-Δ32 homozygotes in the general population and the difficulties of identifying suitable donors, alternative methods to artificially disrupt CCR5 are being sought. RNA interference has successfully been used to silence CCR5 as well as viral genes (reviewed in [12]); but it requires the continuous presence of siRNA, and the gene ablation is never complete. On the other hand, gene-editing methods have attracted a lot of attention as potential therapy for HIV, as they allow permanent disruption of the selected gene(s). 

Targeted gene disruption techniques have been extensively used to create mutant mice that allow functional characterization of any gene of interest. Traditionally, homologous recombination (HR) between a targeted genomic sequence and an engineered DNA targeting vector has been used to modify embryonic stem cells, which are then used to derive mutant mice [13,14,15,16]. However, due to the extremely low frequency of HR, this technique cannot be used for human gene therapy. Recent progress in understanding the DNA repair pathways and engineered nucleases has allowed the development of simple and efficient alternative methods to induce gene modifications. Now it has become clear that following double stranded break, DNA is repaired either by HR (when a homologous DNA template is also provided) or by the error prone non-homologous end joining (NHEJ) pathway, attended with small nucleotide additions or deletions that results in disruption of the reading frame and gene expression. Use of this strategy for targeted gene disruption has been made possible by the development of engineered nucleases. The power of this technology can be gleaned from the fact that it was designated the method of the year in 2011 by Nature Methods [17]. In this review, we will describe the advances made in developing three kinds of site-specific nucleases, namely zinc finger nucleases (ZFNs), transcription activator-like effector nucleases (TALENs), and most recently, the CRISPR/Cas9 system; and discuss their application towards HIV-1 therapy.

## 2. Zinc Finger Nucleases

**Figure 1:**
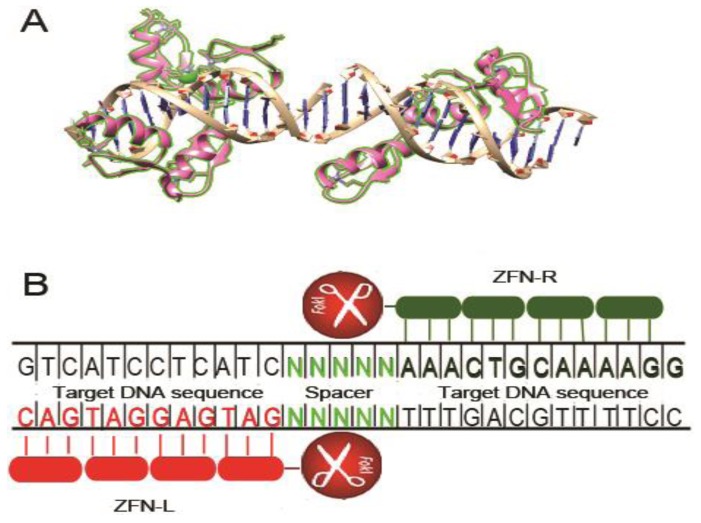
Structure and schematic representation of zinc finger nuclease (ZFN) bound to the target site. (**A**) Crystal structure of a six-finger zinc finger protein (ZFP) complexed with its target DNA fragment (PDB ID: 1MEY, modeled by UCSF chimera). Each single zinc finger consists of one α-helix and two antiparallel β-sheets and contacts DNA with side chains of the α-helix; (**B**) Schematic representation of ZFN bound to double-stranded DNA. Each zinc finger unit recognizes 3 nucleotides of DNA, each ZFN module (left and right) recognizes 9–18 nucleotides. Once both ZFN modules bind DNA, the catalytic domain of Fok I endonuclease dimerizes and cleaves the DNA at the spacer region.

Zinc finger proteins (ZFP) are a versatile class of eukaryotic transcription factors that bind DNA via zinc finger motifs. These DNA binding motifs are linked to the nuclease domain of Fok 1 restriction endonuclease to create zinc finger nucleases (ZFNs). ZFNs thus combine the favorable qualities of both components-the DNA binding specificity and flexibility of ZFPs. The cleavage activity of Fok 1 can only occur after DNA binding, thereby avoiding nonspecific cleavage (Figure 1). The zinc finger domain typically contains three to six zinc finger modules linked together, and each finger module of 30 amino acids recognizes three sequence-specific nucleotides [18]. Therefore, each zinc finger module array recognizes between 9 and 18 base pairs. The Fok I cleavage domain functions as a dimer [19] and gets activated for cleavage after dimerization. Thus, the two zinc finger module arrays are designed to bind to 9–18 nucleotides on opposite sides of the sequence targeted for disruption on the sense and the antisense strand respectively (separated with a spacer of 5–7 nucleotides) to bring the Fok I units together as a dimer, which initiates the cleavage process to generate a double strand break (DSB) at the spacer region. Since each designed ZFN pair specifically recognizes 18–36 base pairs, it can theoretically be designed to make it unique and highly specific for the targeted gene. The only constraint, that has been mostly solved by systematic studies, is that one must know the finger module that recognizes any given (of the 64 possible) nucleotide triplet.

Once the DSB is introduced, it can stimulate the cellular DNA repair mechanisms. The innate DNA repair mechanisms consist of either the error-prone NHEJ [20] or homology directed repair by HR [21]. However, the HR pathway needs an externally provided homologous DNA template; and thus in most circumstances, NHEJ is the predominant repair mechanism for DSBs generated by ZFNs. This repair pathway is attended with nucleotide insertions, deletions or frame-shift mutation [22], leading to gene disruption. 

Taking advantage of the effective and sequence-specific gene modification capability of ZFNs, the technology has been widely utilized for genome editing in various types of cells and living organisms [23,24,25,26,27,28,29]. As for HIV-1 gene therapy by ZFNs, most publications have focused on co-receptor CCR5 or CXCR4 disruption [30,31,32], but targeting HIV-1 genome is a possible alternative strategy [33].

### 2.1. CCR5 Disruption

Theoretically, the cellular receptor or coreceptors involved in HIV-1 entry could be used as targets for inhibiting HIV-1 infection. However, the major viral receptor CD4 plays an important role in the immune system, which makes its deletion unacceptable. On the other hand, individuals with mutations in the HIV co-receptor CCR5 have no major immune deficits [34,35] except for a slight increase in the risk of West Nile infection [36]. Hence, CCR5 disruption is considered an attractive therapeutic target to block HIV-1 entry. Compared to conventional gene knockdown by RNAi, CCR5 disruption by ZFN is heritable and thus, one time treatment can potentially render the cell permanently HIV resistant. 

The first report on CCR5 alteration by ZFN technology was published in 2005 [37]. Mani *et al*. showed that three-finger ZFN was able to specifically cleave CCR5 at the designed site using an *in vitro* transcription-translation system. Since then, many studies have successfully disrupted CCR5 in various cell lines, primary T cells and hematopoietic stem cells (HSCs), as well as in humanized mice [30,31,32,38,39,40]. From the point of HIV-1 therapy, genetic disruption of CCR5 in CD4+ T cells and CD34+ HSCs is most relevant. CD4+ T cells can be gene edited *in vitro* and reinfused into patients to provide a source of HIV resistant T cells. Theoretically, CD34+ hematopoietic cells can be rendered CCR5- *in vitro* and infused to patients, so that the progeny T cells and macrophages provide a continuous source of HIV resistant cells. Perez and colleagues demonstrated that ZFNs can permanently and specifically disrupt CCR5 in 50% of primary CD4+ T cells with minimal off target effects (<5% at CCR2 loci). After *ex vivo* expansion, the modified CD4+ T cells were engrafted into NOG mice. Gene edited CD4+ T cells effectively reconstituted and provided significant resistance to subsequent HIV infection [30]. Moreover, following infection, the gene modified T cells were substantially enriched, probably because HIV killed the resident unmodified but not the modified T cells. The same group has recently scaled up the process and was able to derive 10^10^ gene modified CD4+ T cells by using AD5/F35 adenoviral vector for transduction and anti-CD3/anti-CD28 stimulation for expansion of CD4+ T cells [41]. Moreover, the CD4+ T-cell phenotype, cytokine production, and repertoire were comparable between ZFN-modified and control cells. Further, infusion *in vivo* into NSG mice was not associated with any toxicity or T cell transformation. Based on these results, the group has recently initiated a clinical trial for infusion of gene modified CD4 T cells in HIV infected individuals. (ClinicalTrials.gov, phase I by University of Pennsylvania, NCT00842634). A similar clinical trial has also been initiated by Sangamo Biosciences, (NCT01252641 and NCT01044654). Early results suggest that the infused gene modified T cells are harmless, persist over time, and traffic to different organs. However, the real efficacy can only be tested by interruption of ART, which is hard to do because of ethical and regulatory concerns.

ZFN-mediated CCR5 gene modification has also been achieved in hematopoietic stem cells. Yao *et al*. showed that ZFN was able to efficiently and precisely disrupt CCR5 in embryonic and induced pluripotent stem cells (ESCs and iPSCs), and that these cells could be differentiated into CD34+ HSCs [42]. Holt and co-workers used nucleofection to achieve ZFN-mediated CCR5 disruption in human CD34+ HSCs. Transplantation of gene modified HSCs in NSG mice resulted in multi-lineage reconstitution of human immune cell types. Moreover the reconstituted cells showed resistance to HIV infection. Similar to CD4+ T cells engraftment described earlier, the gene-modified cells were greatly enriched after infection [43]. Likewise, Lambardo and colleagues also disrupted CCR5 using a non-integrating lentiviral vector-delivered ZFN in HSCs [44], and AD5/35 delivered ZFN in primary T cells, human neural stem cells (hNSCs) and in iPSCs [45]. Most recently, Li and co-workers modified CCR5 at a ratio more than 25% in adult HSCs (HSCs isolated from adult blood after mobilization with GMCSF) by using adenoviral vector-delivered ZFN, assisted with optimized dose of protein kinase C activator. The modified adult HSCs could also be reconstituted and were fully functional in NSG mice [46]. 

### 2.2. CXCR4 Disruption

Generally, CCR5 serves as the predominant co-receptor for HIV entry during initial transmission and through the early stages of infection. However, once HIV-1 infection is established, it is able to choose CXCR4 as an alternative co-receptor [47,48]. With the emergence of CXCR4 tropic viruses, CCR5 disruption will no longer be able to protect against the spread of HIV-1. For this reason, targeted CXCR4 disruption is also being considered as an additional strategy for inhibiting HIV-1 infection. To date, three studies have demonstrated that targeting CXCR4 can promote HIV-1 resistance. The Doms group at the University of Pennsylvania was the first to engineer CXCR4 deficient CD4+ T cells by CXCR4-ZFN delivery via AD5/35. The modified CD4+ T cells proliferated normally and showed resistance to CXCR4 tropic HIV-1 infection *in vitro*. When the modified CD4+ T cells were transplanted into NSG mice, the mice showed resistance to X4 HIV-1. However, the protection failed because of the emergence of CCR5-tropic virus mutants over time [31]. Similarly, Yuan and co-workers compared the efficiency of X4 targeting shRNAs and ZFN, and found that ZFN gene disruption was superior to shRNA in conferring protection against X4 tropic virus. They also verified that CXCR4-ZFN disrupted CD4+ T cells conferred resistance in the humanized mouse model [32]. As these studies collectively show, disruption of CXCR4 or CCR5 alone may not be sufficient to provide stable protection as they could potentially force the emergence of the respective X4 or R5 tropic HIV-1 variants. To overcome this limitation, a novel strategy has been used by Voit and colleagues [39]. They used ZFN to incorporate three restriction factors, TRIM5α, APOBEC3G and D128K, or Rev M10 at the CCR5 locus, thereby knocking out CCR5 as well. The modified T-cell lines were robustly resistant to both R5 tropic and X4 tropic HIV-1. This approach may be better than the dual co-receptor knockout strategy because viral blockade occurs at multiple steps. Further, the CXCR4 receptor plays a physiologically important role, raising concerns that its deletion *in vivo* may have functional consequences.

### 2.3. Targeted HIV-1 Proviral DNA Disruption

As described above, disruption of CCR5 and CXCR4 can only stop the spread of new virus, which could eventually result in a functional cure. However, this will not be sufficient to eradicate the virus from already infected cells. Moreover, disruption of either gene opens up the possibility of generating alternative co-receptor using mutants [33]. To reach the ultimate goal of a real cure, it is necessary to eradicate the proviral DNA that is already integrated into the host genome in the infected (including latently infected) cells. Therefore, attempts have also been made to eliminate HIV proviral DNA using ZFN technology.

ZFN technology has also been used to successfully target proviral DNA in HBV [49], HSV-2 [50] and HTLV-1 [51] infection. These proof-of-concept studies suggest that it is also possible to target HIV proviral DNA. By using a computational model, Wayengera reported that an 18 bp sequence from the HIV-pol gene could induce specific gene disruption. They also used ZFN to target various sites within the proviral DNA and found the ZFN pairs that could delete ~80% of proviral DNA [52]. Das *et al*. designed and screened three ZFNs which could efficiently cleave the conserved regions of HIV-1 genes gag, pol and rev [53]. Most recently, the Zhu group in China used a specially designed ZFN to target the highly conserved 5′ and 3′ LTR sequences of HIV-1 to successfully eradicate HIV-1 proviral DNA in cell lines as well as in acutely infected and latently infected primary T cells [33]. Although these results are exciting, a number of hurdles must be overcome for targeting the HIV genome in a clinical situation. For example, patients harbor the integrated HIV-1 provirus in resting T cells, and the latently infected cells are extremely rare (~only one in 1 million CD4+ T cells), so delivery of ZFN to these cells in vivo will pose a major challenge. Nevertheless, the proof-of-principle preliminary studies described above demonstrate that targeting the provirus is a potential strategy for curing HIV infection.

### 2.4. ZFN Delivery Strategies for HIV Gene Therapy

Delivery of gene therapy vehicles to specific target cells has been a major bottleneck for translation to human therapy. Gene therapy for HIV infection is mainly focused on CD4+ T cells or CD34+ HSC. Because of the difficulty in targeting these cells *in vivo*, the general approach is to modify the cells *in vitro* with the idea that they may be used for reinfusion to infected subjects. With the current state of technology, gene therapy for HIV is generally intended mainly for use in already infected people. 

As a proof-of-concept delivery method, an adenoviral vector was used to deliver ZFNs to activated CD4+T cells *in vitro*, reaching a gene-disruption efficiency as high as 54% for CCR5 [30] and 30%–34% for CXCR4 [31,32]. After expansion *in vitro*, the adenovirus-ZFN transduced CD4+ T cells were infused into humanized mice where they were able to establish resistance to HIV-1 infection. For CD34+ cells, ZFN-AD5 vector also achieved >25% CCR5 disruption [46]. Due to the maturity of techniques for generating high titer adenovirus stocks [54,55], it has also been scaled up for clinical trials [41]. Although one common concern in using adenovirus delivery *in vivo* is the pre-existing immunity that might neutralize the virus, making delivery ineffective, this should not be a problem for delivery *in vitro*. However, several studies show that adenovirus can increase the susceptibility to HIV-1 infection [56,57]. Non-replicating HIV-based lentiviral vector is also a versatile tool for ZFN delivery for HIV-1 therapy. By changing the envelope gene from HIV to VSV, the resulting packaged lentivirus can transduce various types of cells in the immune system that are associated with HIV-1 infection. However, continued long-term expression of ZFN from integrated lentiviral vector can increase the chances of off target effects. As ZFN genome editing is heritable, long-term expression is not needed. Hence the toxic effects can be mitigated by using mutant, non-integrating lentivirus for transient expression of ZFN. With this in mind, Lombardo *et al*. used a non-integrating lentivirus to deliver ZFN to disrupt CCR5 in CD34+ HSCs. They were able to disrupt CCR5 in HSCs, though the efficiency was relatively low (5%) [44]. In addition baculoviral vector has also been used to transduce hES cells to achieve a 5% efficacy of CCR5 disruption [58]. 

Compared to viral delivery vectors, non-viral methods will likely be more cost effective and have minimal safety concerns. Thus, plasmid transfection by nucleofection has been used for ZFN-mediated CCR5 disruption. This method resulted in 17% CCR5 disruption in HSC. When these cells were transplanted into NSG mice, the relatively low number of modified cells selectively expanded after *in vivo* HIV challenge [43]. mRNA electroporation also provides a means for transient expression to allay safety concerns and thus, CCR5-ZFN mRNA has also been recently used. Using the MaxCyte GT system (that can treat 300 million cells at a time, allowing clinical scale usage), Cannon *et al*. achieved an efficiency of CCR5 gene disruption ranging from 30%–50% in patient derived-HSCs. After engraftment into NSG mice, these cells supported multilineage reconstitution of human immune system, and the gene modified cells persisted even after 6 months of reconstitution [59]. Another exciting development in this regard is the finding by Gaj *et al*. that the ZFP has an intrinsic cell-penetrating property that allows entry of the protein into cells without need for a delivery vehicle. They recently incubated CD4+ T cells with recombinant CCR5-ZFP protein and found that this led to gene disruption, although the efficiency was in the lower range of ~8% [60]. Taken together, these studies have shown that non-viral delivery of ZFN is a feasible tool for HIV therapy, although further improvements may be required. 

### 2.5. Specificity of ZFN Targeted Gene Disruption in HIV-1 Therapy

The fundamental safety concern for using ZFN mediated gene disruption for human therapy is the specificity of gene targeting. For example, if the nuclease action mutates unintended targets that are important for cellular physiology, the treated cells may become dysfunctional or even die [61,62,63]. Therefore, it is essential that ZFNs be carefully designed to avoid off-target cleavage events as far as possible. 

For CCR5 disruption, the major concern comes from its homologous locus CCR2, because of their high sequence similarity. Kim *et al*. found that CCR5 ZFN induces off-target activity within CCR2 gene even when there is a single mismatch between the targeted CCR5 sequence and CCR2 DNA [40]. Perez *et al*. found that with two mismatches, the cleavage activity for CCR5 is 10-fold higher than for CCR2 [30]. In an interesting study, Pattanayak *et al*. found that high-affinity binding of one CCR5-specific ZFN monomer in a pair can compensate for another monomer's weak binding [64]. Thus, tight binding (complete homology) of one half can cause off target effects even if the other half contains two-three mismatches, showing the importance of designing ZFNs with less binding energy at both arms to avoid off-target effects. Moreover, they also showed that concentration of ZFN is an important factor for off-target cleavage, suggesting that concentration of ZFN should be as low as possible [64]. Another study used an integrase-deficient lentivirus capture (IDL genome gets inserted at DSBs created by ZFN) assay to measure genome wide integration as a way to detect off-target effects in an unbiased manner. The results were also confirmed by deep sequencing to detect ZFN-induced indels. This analysis suggested that although off-target cleavage occurred only at consensus stretch of DNA, cleavage did not occur at all loci showing consensus predicted *in silico* [65]. Taken together, these studies highlight the fact that proper evaluation of the specificity of any particular ZFN pair will require the use of the relevant cell type, the intended ZFN dose and the delivery method for that specific application. This will be important to avoid potential failures in clinical trials.

## 3. Transcription Activator-like Effectors Nucleases (TALENs)

Transcription activator-like effectors (TALE) are naturally occurring DNA binding proteins from plant bacterial pathogen, Xanthomonas. TALE proteins contain N and C termini for localization and activation and a central domain for specific DNA binding. The central domain contains a variable number (5–30, average 15.5) of tandem monomer repeats that confer DNA binding specificity. The discovery that TALEs use a simple modular code for DNA recognition has provided an alternative platform for genome editing and gene disruption. Similar to ZFN, a pair of custom TALE DNA binding domains fused to Fok I endonuclease (binding to opposite sides of target DNA with a spacer of 14–18 nt to allow dimerization of Fok 1 and cleavage of DNA) is used for gene editing. In a short time after discovery, this technique has already been widely used for genome editing in a variety of cells, plants, model organisms, livestock and even human cells [66,67]. 

**Figure 2:**
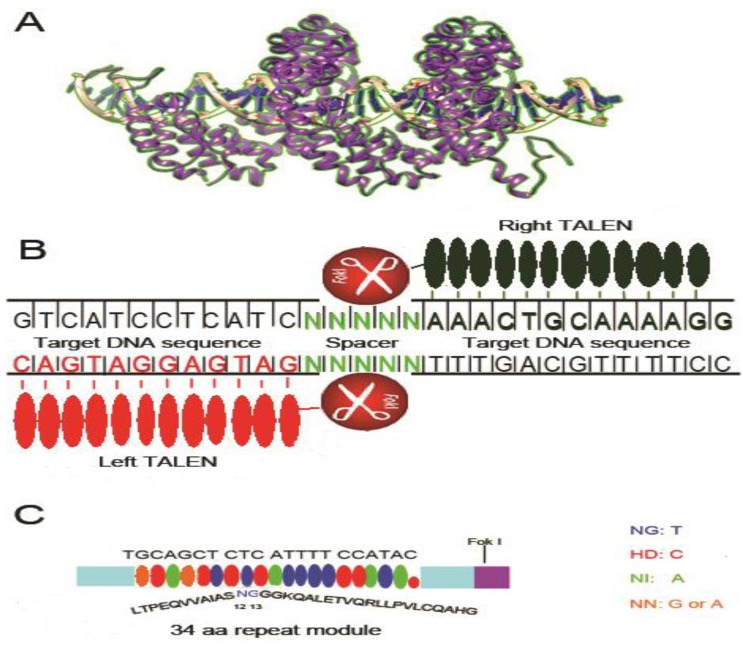
Structure and schematic representation of transcription activator-like effector nucleases (TALEN) bound to the target site. (**A**) Crystal structure of a TALEN complexed with the target DNA fragment (PDB ID: 3UGM, modeled by UCSF chimera software). Each single TALE unit is composed of two similar α-helices with intervening two variable amino acids that specifically recognize one single nucleotide; (**B**) Schematic representation of TALEN bound to double stranded DNA. Each TALEN unit recognizes one single nucleotide. Once both TALEN modules bind DNA, the Fok I endonuclease dimerizes and cuts DNA at the spacer region; (**C**) TALEN nucleotide recognition code. Variable diresidues (RVDs) located at amino acids 12 and 13 of each TALEN unit recognizes a single nucleotide according to the code: NG for T, HD for C, NI for A and NN for G or A.

Unlike ZFN, where each finger module (30 amino acids) recognizes three nucleotides, each TALE repeat consists of 33–35 (34 in most cases) amino acids and recognize only one nucleotide [68] (Figure 2). The DNA recognition specificity is contributed by the highly variable amino acids at position 12 and 13, so called repeat variable diresidues (RVDs) (for example, HD (His, Asp) targets cytosine (C), NI (Asn, Ile) targets adenine (A), NG (Asn, Gly) targets thymine (T), and NN (Asn, Asn) targets guanine (G) and adenine (A)). Especially, the last repeat called “half repeat” contains only the first 20 amino acids of the complete repeat [68,69]. After RVDs bind the cognate nucleotide sequences, the Fok I domains on opposite pairs of TALEN dimerize and cut the DNA at the spacer region to generate DSB. Repair of DSBs via NHEJ pathway commonly results in indels with nucleotide deletions, insertions resulting in frame shift and gene disruption. Alternatively, TALEN mediated DSBs can also stimulate homologous recombination in the presence of homologous donor DNA, enabling site-specific insertion of an exogenous sequence. Due to the simple “protein-DNA code” TALENs can be easily designed and constructed to bind any unique sequence in the genome. TALE modules that recognize 14–20 target nucleotides are generally used for gene editing. TALE repeats recognizing particular nucleotides are assembled together for this purpose. However, due to the high sequence similarity between each repeat of TALENs (only two RVD residues differ between modules of 34 aa each), it is a challenge to assemble them together in the same construct. 

Numerous studies have been grappling with the problem of assembling the TALEN repeat arrays and a variety of cloning techniques have been employed to overcome this difficulty. The first method for assembling TALEN arrays is restriction enzyme and ligation cloning (REAL), in which the single repeats are connected together via normal digestion-ligation method [70,71,72]. By using type IIS restriction enzymes to generate orthogonal ligation linkers between each repeat, the Golden Gate cloning method has accelerated the cloning process of TALENs, in which the repeats can be assembled quickly in a hierarchical way [73,74,75,76,77]. Furthermore, the high-throughput solid-phase ligation strategy provides the possibility of large-scale and cost effective synthesis of TALENs [78,79]. More recently, ligation-independent cloning technique has been utilized for high-throughput assembly of TALEN genes. The high fidelity of the overhangs of each repeat yields higher ligation efficiency and specificity as well as makes synthesis much simpler for systematic studies on genomic research [80].

So far, the only attempt using the TALEN system for HIV-1 gene therapy was reported in 2011 [81]. Mussolino and co-workers compared CCR5 gene disruption using ZFN and TALEN side by side in 293 T cells. Comparable levels of gene disruption (~45%) could be achieved with both approaches. However, TALEN demonstrated much lower cytotoxicity and significantly reduced off-target activity at the CCR2 locus. Although these results are promising, further studies demonstrating efficacy in humanized mouse models of HIV infection are required to validate this technology. The relatively large size of the protein (compared to ZFN) is a hurdle for delivery that must be overcome. However, although attempts to express TALEN in lentiviral vectors have failed, it has been expressed successfully in adenoviral vectors [82]. Genome wide off-target effects will also need to be determined carefully to ensure safety.

## 4. Engineered CRISPR/Cas9 System

**Figure 3:**
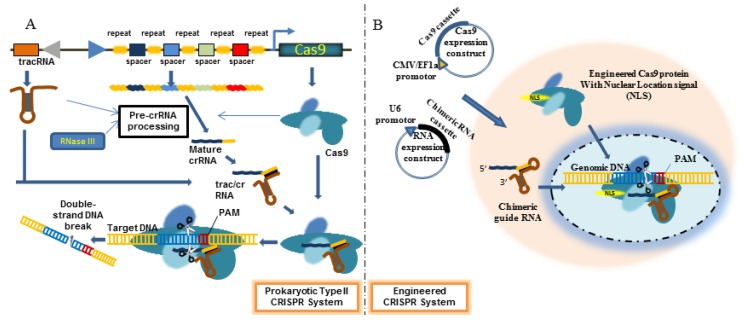
Schematic representation of clustered regularly interspaced palindromic repeats (CRISPR)/Cas9 System. (**A**) In prokaryotic cells, the CRISPR locus contains an array of repeats (of the same sequence) interspersed with spacers with unique sequences. This locus also contains DNA coding for tracRNA and Cas proteins. The transcribed pre-crRNA is processed by RNAse III into mature crRNA that associates with tracRNA. This RNA duplex then binds the Cas9 protein to form a ribonucleotide complex. crRNA guides this complex to bind complementary (to spacer in crRNA) target DNA following which, the Cas9 protein cleaves the DNA to induce DSB; (**B**) In engineered CRISPR/Cas9 system, a plasmid encoding a chimeric RNA consisting of crRNA targeting the gene of interest fused at its 3' end to tracrRNA and a plasmid encoding the Cas9 protein are cotransfected.

Bacterial genomes contain loci encoding what is known as clustered regularly interspaced palindromic repeats (CRISPR), consisting of an array of short direct repeats of 21–47 nts (average 32) interspersed with short intervening spacers (Figure 3). While the repeats are identical, the spacers vary in sequence. The CRISPR locus is surrounded by a cohort of CRISPR-associated (Cas) genes. The transcribed CRISPR RNA (crRNA) products associate with Cas protein/nuclease and guide the complex to the target DNA that is complementary to the spacer sequence, whereupon the Cas protein cleaves the DNA to create DSB followed by DNA repair by NHEJ or HR as described for ZFN and TALEN [83]. This pathway is commonly used by bacteria to destroy foreign invaders (like plasmids and phages), and to evade host innate immunity to enhance bacterial virulence [84,85]. This system has recently been shown to have enormous potential for gene editing in a variety of hosts including human cells.

There are several types of CRISPR/Cas system in diverse bacterial species. They all involve a few small, non-coding CRISPR RNAs and a set of Cas proteins. Type II CRISPR/Cas system uses less components than other types. Here, a small CRISPR RNA (crRNA consisting of sequence-specific spacers flanked by repetitive elements) and a partially complimentary trans-acting RNA (tracRNA) is all that is necessary for recognition of target DNA. A single Cas9 protein associates with crRNA and tracRNA to mediate target DNA degradation. A significant recent advance for using CRISPR/cas system for artificial gene editing was the demonstration that crRNA and tracRNA could be engineered as a single RNA chimera (called guide or gRNA) that leads to sequence-specific dsDNA cleavage by Cas9 [86]. The short chimeric crRNA/trac RNA can be easily expressed using a pol III promoter (such as U6) and Cas 9 can be expressed in a separate plasmid under CMV or EF-1α promoter. This innovation enables easy gene editing with custom synthesized gRNA that can be used to target any host gene. The only prerequisite for the DNA to be a CRISPR target site is that it needs to be preceded by a NGG sequence (protospacer adjacent motifs, PAM). In fact, in less than a year of discovery, engineered CRIPSR/Cas9 system has been used for gene editing in most biologic models including Saccharomyces cerevisiae, C. elegans and a variety of mouse and human cells. 

Compared with other genome editing tools such as ZFN and TALEN, CRISPR/Cas 9 system holds many obvious advantages as well as potential limitations. Unlike ZFN and TALEN, CRSPR/Cas9-mediated gene editing system adopts a Watson-Crick complementarity rule via a short RNA molecule that is homologous to the target site. Since Cas-9, which is the common moiety is already available in a cloned form, all that one needs is to synthesize and express a short RNA for any gene of interest. Thus, a major advantage compared to other methods that usually require labor-intensive design and screening, is that the CRSPR/Cas9 requires much simpler design and a single cloning step. For almost any lab throughout the world, a versatile gene-editing tool is literally a few oligo synthesis away. 

Another obvious advantage of CRISPR/Cas9 system is its high versatility. As part of the adaptive immune system, it has a natural propensity to target multiple gene locations simultaneously. As described by Zhang group [87], CRISPR/Cas 9 system can be easily employed to target multiple sites to induce a deletion of a large gene fragment. Moreover, although Cas9 protein naturally induces double stranded DNA breaks by cutting each strand with two separate motifs, a mutation within one of the enzymatic motifs of Cas9 converted it into a valuable nickase with the capability to induce homologous recombination and the mutant is much safer to use with less off-target effects [88]. Furthermore, a Cas9 mutant totally defective in endonuclease activity together with a guide RNA can specifically interfere with gene transcription without inducing any gene mutation in a process called CRISPR interference (CRISPRi) [89].

Efficacy of gene disruption is one of most important criteria for a gene-editing tool. In the few available publications using CRISPR/Cas9 system, the gene editing frequency is comparable with ZFN or TALEN [87]. On the other hand, off-target effects of engineered CRISPR/Cas9 system are far from clear, but are being increasingly studied. An early report indicated that even a single mismatch within 13–14 bp of the 3'-terminal crRNA sequences (5' to PAM site) abolishes the cleavage activity, suggesting a high degree of specificity and less off-target effects. Moreover, no significant cytotoxicity was observed [87,88]. However, it has been recently shown that CRISPR/Cas9 system can be highly active even when mismatches occur between gRNA and their DNA targets, and off-target mutations occur at endogenous loci in human cells with high frequency [90,91,92]. Collectively, these studies indicate that although each base within the 20 nt guide sequence contributes to overall specificity, multiple mismatches between the guide RNA and its complementary target DNA sequence can be tolerated depending on the quantity, position, and base identity of mismatches [87,90,91,93], all of which contribute to potential off-target activity. Considering these variables, an online design tool has been described [94] that helps avoid such off-target effects. Since the off-target cleavage occurs in a CRISPR/Cas9 dose-dependent manner, limiting the amount of Cas9 and gRNA plasmids delivered may be another way to minimize off-target effects. 

An important recent advance to minimize off-target effects is the use of double nicking strategy [95]. While wild type Cas9 cuts both strands of DNA, it can be converted into single stranded DNA nickase by mutating one of the cleavage domains. Compared to DSB, which is repaired by error prone NHEJ pathway, single stranded nicks are repaired by error free base excision repair (BER) pathway. In the double nicking strategy, a pair of gRNAs targeting adjacent areas on the opposite strands of target DNA are used along with the mutant Cas9 nickase. While this induces double stranded breaks in target DNA, it is expected to induce only single stranded nicks in off-target genes (because of rarity of occurrence of two adjacent off-target sites) that get repaired by error free BER pathway. Indeed, a systematic comparison of on and off-target cleavage showed that the double nickase system had 50–1500 fold decreased off-target activity while maintaining on target cleavage comparable to wild type Cas9. 

CCR5 gene is also an ideal target for engineered CRISPR/Cas9 nuclease editing, similar to ZFN and TALEN systems. Kim and colleagues made the first attempt in this direction [96]. In their study, co-delivery of Cas9 expression construct with gRNA encoding plasmid into human cells induced up to 18% mutation at the CCR5 locus. In another study, CRISPR/Cas9 system was used to target HIV-LTR. This proof-of-principle study showed that the LTR targeting system could excise the integrated proviral DNA, providing a way to eliminate HIV latency [97]. In unpublished studies, we have found that gRNAs can be selected to induce up to 50% gene editing at the CCR5 locus in 293 T, as well as in Jurkat cells. Moreover, we have also successfully expressed both Cas9 and gRNA in a lentiviral vector for use in difficult to transfect primary T cells. Since this system can also be used to target multiple regions to induce deletions, one potential application for HIV would be to eliminate the integrated proviral DNA as a strategy to eradicate reactivation from latently infected cells. 

Considering that it has been barely a year since first reported, in future studies CRISPR/Cas9 system will very likely be improved for both efficiency and reduced off-target effects. We can expect that new delivery methods will also be discovered in the next few years. 

## 5. Conclusion and Future Directions

ZFNs, TALENs and CRISPR/Cas9 are versatile tools for gene disruption that have the potential for revolutionizing the field of HIV gene therapy. They provide powerful means to disrupt CCR5 and other host factors necessary for HIV infection as well as to delete the integrated HIV genome as a means to eradicate HIV latency. Towards this end, great progress has already been made with ZFN; which has been evaluated in humanized mouse models, and is being tested in clinical trials. TALEN and CRISPR/Cas9 systems, which are relatively new, will undoubtedly be similarly evaluated soon. However, for actual use in HIV-1 gene therapy, there are many challenges that have to be overcome. First of all, the delivery of gene-editing mediators needs to be optimized for relevant cell types. Although *in vitro* transfection and lentiviral and adenoviral vectors have been successfully used to deliver ZFNs into human cells, delivery of the larger sized TALEN is likely to be a challenge. Secondly, for a chosen gene editing method, the off-target effects need careful evaluation at the genome wide level. The rapid application of high-throughput analysis via deep sequencing methods offers hope that this problem will be soon solved. In addition, cytotoxicity of the endonuclease for primary T cells and CD34+ cells needs to be studied in detail. Toxicity and off-target effects can potentially be reduced by transient expression using methods such as directly introducing ZFN protein, mRNA transfection or expression via non-integrating lentiviral vectors. In summary, the rapid progress in the development of newer gene editing technologies offers hope for using these technologies for actual HIV gene therapy in the near future. 
